# Somatostatin slows Aβ plaque deposition in aged *APP*^*NL-F/NL-F*^ mice by blocking Aβ aggregation

**DOI:** 10.1038/s41598-023-29559-z

**Published:** 2023-02-09

**Authors:** Declan Williams, Bei Qi Yan, Hansen Wang, Logine Negm, Christopher Sackmann, Claire Verkuyl, Vanessa Rezai-Stevens, Shehab Eid, Nimit Vediya, Christine Sato, Joel C. Watts, Holger Wille, Gerold Schmitt-Ulms

**Affiliations:** 1 Tanz Centre for Research in Neurodegenerative Diseases, University of Toronto, Toronto, ON Canada; 2 Department of Laboratory Medicine & Pathobiology, University of Toronto, Toronto, ON Canada; 3 Department of Biochemistry, University of Toronto, Toronto, ON Canada; 4 Centre for Prions and Protein Folding Diseases, University of Alberta, Edmonton, AB Canada; 5 Department of Biochemistry, University of Alberta, Edmonton, AB Canada; 6 Tanz Centre for Research in Neurodegenerative Diseases, University of Toronto, Rm 6KD447, 60 Leonard Ave, Toronto, ON M5T 0S8 Canada

**Keywords:** Neuroscience, Molecular neuroscience

## Abstract

The neuroendocrine peptide somatostatin (SST) has long been thought of as influencing the deposition of the amyloid β peptide (Aβ) in Alzheimer’s disease (AD). Missing have been in vivo data in a relevant Aβ amyloidosis model. Here we crossed *App*^*NL-F/NL-F*^ mice with *Sst*-deficient mice to assess if and how the presence of Sst influences pathological hallmarks of Aβ amyloidosis. We found that Sst had no influence on whole brain neprilysin transcript, protein or activity levels, an observation that cannot be accounted for by a compensatory upregulation of the Sst paralog, cortistatin (Cort), that we observed in 15-month-old Sst-deficient mice. Sst-deficiency led to a subtle but significant increase in the density of cortical Aβ amyloid plaques. Follow-on western blot analyses of whole brain extracts indicated that Sst interferes with early steps of Aβ assembly that manifest in the appearance of SDS-stable smears of 55–150 kDa in *Sst* null brain samples. As expected, no effect of Sst on tau steady-state levels or its phosphorylation were observed. Results from this study are easier reconciled with an emerging body of data that point toward Sst affecting Aβ amyloid plaque formation through direct interference with Aβ aggregation rather than through its effects on neprilysin expression.

## Introduction

The peptide hormone somatostatin (SST) was initially discovered as an inhibitor of growth hormone secretion^1,2^. It is now understood that SST, along with its paralog cortistatin (CORT)^3^, modulates a host of activities in the gastrointestinal, nephritic, immune, and central nervous systems^4^. Both hormones are released from larger proproteins exceeding one hundred amino acids in length through endoproteolytic cleavages of C-terminal peptides which cyclize by disulfide bonding. Alternative endoproteolytic processing produces prominent variants named according to their amino acid sequence lengths as SST-14, SST-28, CORT-17, and CORT-29. These peptides have similar yet distinct physicochemical properties and affinities for five human somatostatin receptors (SSTRs), which they bind to following their release into the extracellular space^5–8^. The distinct binding profiles of SST and CORT, in turn, elicit distinct physiological responses that point toward overlapping but non-identical functions^9–11^.

Separate lines of biochemical, histological, and genetic evidence implicate the somatostatinergic system in Alzheimer’s disease (AD)^12–14^. In fact, among the first biochemical analyses of postmortem AD brains were two studies that drew attention to a profound reduction in somatostatin levels relative to age-matched control brains^15,16^, a finding that has since been validated in human postmortem brains^17^ and an AD mouse model^18^.

Somatostatin-expressing neurons or interneurons may be more vulnerable to certain AD-associated stressors. For instance, cortical SST immunoreactive neurons were reported to be more susceptible to degeneration than neighboring neuropeptide Y expressing cells, with the loss of these neurons proportional to amyloid plaque and neurofibrillary tangle burden^15,19^. Similarly, somatostatinergic interneurons of the temporal cortex, but not the hippocampus or entorhinal cortex, are disproportionately lost in AD^20^, compared to parvalbumin interneurons, which resist Aβ-induced toxicity in AD patients and mouse models^20,21^.

One way to rationalize these observations is to note that amyloid plaques and distressed somatostatinergic neurons are often found in close proximity, pointing toward a scenario of somatostatinergic neurons increasing the propensity for Aβ to form aggregates nearby^22,23^. A possible causal relationship has also been raised by genome-wide association studies. More specifically, the same single-nucleotide polymorphism in the 3′ untranslated region of the *SST* gene was associated with increased late-onset AD risk in two genetically separate population cohorts in Finland and China, particularly among individuals carrying the apolipoprotein E-ε4 allele^24,25^.

What might be the mechanism through which SST modulates AD?

SST was initially reported to promote Aβ clearance indirectly. Thus, in mouse cortical neuronal cultures, a modest decrease in extracellular Aβ_42_, but not Aβ_40_, followed a single administration of nanomolar to low micromolar SST-14. This effect was attributed to an SST receptor-dependent transcriptional activation of neprilysin^26^, a protease that the same group had previously shown to digest Aβ^27^. SST has also been shown to enhance the proteolytic activity of insulin degrading enzyme (IDE) toward Aβ in vitro, to increase microglial IDE transcription and translation, as well as phagocytosis of Aβ^28^, and to be an IDE substrate itself^29,30^.

More recently, a direct influence of SST on Aβ emerged when we identified SST by mass spectrometry as the smallest human protein that interacts selectively with oligomeric but not monomeric Aβ^31^. We then established that nanomolar concentrations of SST or CORT modulate the aggregation of Aβ_42_^31^. Since then, the selective SST binding to oligomeric Aβ_42_ has been shown to extend to a β-sheet pore-forming Aβ_42_ tetramer with putative neurotoxicity^32^. The possibility that SST may modulate Aβ plaque formation directly is heightened by the fact that this molecule spontaneously forms amyloid fibrils in vitro^33^ and is, in fact, stored prior to its synaptic release in dense granules as a natural amyloid^34^. Upon their release, it takes in the order of minutes to hours before these SST amyloids are dissolved^35^, providing an opportunity to influence nearby Aβ aggregation in AD^12^.

Remarkably, despite all these observations implicating somatostatin as a possible factor in AD spanning decades, no prior experimental study has investigated the influence of SST in an in vivo Aβ amyloidosis paradigm. Here, we set out to begin to address this unmet need by studying intercrosses between *Sst* null mice and a humanized *App* knock-in mouse line that is known to develop a profound Aβ amyloidosis as the mice age. Breeding the cross as *Sst* hemizygotes provided progeny of mixed *Sst* genotype which consistently developed Aβ amyloid plaques from middle to late adulthood. We present data that queried how *Sst* deficiency affected (i) transcript levels of cortistatin and neprilysin, (ii) Aβ amyloid plaque burden and plaque distribution, (iii) neprilysin protein and activity levels, (iv) SDS-stable Aβ oligomers, as well as (v) steady-state tau levels and tau phosphorylation. To generate these data, we made use of *App*^*NL-F/NL-F*^*Sst*^*−/−*^ mice, with age-matched Sst-expressing *App*^*NL-F/NL-F*^*Sst*^+*/*+^ mice serving as controls.

## Materials and methods

### Mouse models

*App*^*NL-F*^ mice^36^ were generously provided by Drs. Takashi Saito and Takaomi C. Saido of the Laboratory for Proteolytic Neuroscience, RIKEN Brain Science Institute, Hirosawa, Wako-shi, Saitama, Japan. The B6N.129S4(129S6)-Sst^tm1Ute^/J Sst knockout mouse line^37^ was supplied by the Jackson Laboratory (catalog number 008117, Bar Harbor, ME, USA).

Double transgenic animals of all three *Sst* genotypes were housed together with no more than 5 animals per cage. Water was offered to the mice ad libitum and their feed was 18% protein chow. The cages were kept at elevated room temperature (24 °C) in an environment that was subjected to an artificial twelve-hour day and night cycle. Daily health checks for activity and overall appearance of the mice were conducted. The cages were changed once a week. No behavioral studies were undertaken. At 12 and 15 months of age, the mice were sacrificed exclusively for brain collection. To this end, the mice were deeply anesthetized by isoflurane inhalation and exsanguinated by two-minute transcardiac perfusion with phosphate buffered saline. Upon extraction, each brain was separated at the midline; then the right and left hemispheres transferred to neutral buffered 10% formalin (Sigma-Aldrich, St. Louis, MO, USA) and dry ice, respectively. Right cerebral hemispheres were transferred to 70% ethanol (v/v) after 48 h of fixation, left hemispheres were stored at − 80 °C. Littermates of different *Sst* genotypes were sampled wherever possible.

### Genotyping

Genomic DNA was isolated from tail clips collected at weaning. After overnight Proteinase K (Bioshop, Burlington, ON, Canada) digestion at 55 °C, genomic DNA was isolated by phenol extraction then precipitated and purified with ethanol. The following primers were used to inform the mouse breeding program and to determine *Sst* genotypes of experimental animals: *App* wild-type forward (5′-ATCTCGGAAGTGAAGATG-3′) and reverse (5′-GTTAAGTTCTCATCTACA-3′), *Sst* wild-type (5′-TCAGTTTCTGCAGAAGTCTCTGGC-3′) and knockout (5′-ATCCAGGAAACCAGCAGCGGCTAT-3′) forward and Sst reverse (5′-GAATGCCAATAGTTTGCGCAGCTCC-3′). *Sst* genotyping was performed in accordance with the supplier’s protocol. A second forward primer used to confirm the *Sst* deficient allele was 5′-AGGCACGATGGCCGCTTTGG-3′.

### Antibodies

Monoclonal 4G8 anti-APP/Aβ, applied at 1:2000 dilution (catalog number 800701), and monoclonal 6E10 anti-APP/Aβ, applied at 1:1000 (catalog number 803001), were from BioLegend (San Diego, CA, USA). Monoclonal 82E1 anti-Aβ, applied at 1:1000 dilution was from Immuno-Biological Laboratories (catalog number 10323, Minneapolis, MN, USA). Monoclonal Tau5 anti-Tau (catalog number AHB0042), applied at 1:5000 dilution, monoclonal AT8 anti-Ser202/Thr205-phospho-Tau (catalog number MN1020), applied at 1:1000 dilution, and AT180 anti-Thr231-phospho-Tau (catalog number MN1040), applied at 1:1000 dilution, were sourced from Thermo Fisher Scientific (Mississauga, ON). Anti-neprilysin (catalog number AF1126), applied at 1:1000 dilution, was from Bio-Techne (Minneapolis, MN, USA). Horseradish peroxidase-conjugated horse anti-mouse IgG (catalog number 7076), applied at 1:5000 dilution, was from Cell Signaling Technology (Danvers, MA, USA).

### Western blot analyses

Frozen left cerebral hemispheres of mice were weighed and homogenized with 0.7 mm diameter zirconia beads (catalog number 11079107, BioSpec Products, Bartlesville, OK, USA) in a buffer composed of 100 mM Tris–HCl, pH 8.3, 100 mM NaCl, 2 × Roche PhosSTOP phosphatase inhibitor cocktail (catalog number 4906837001, Roche, Basel, Switzerland), 2 × Roche cOmplete protease inhibitor cocktail (catalog number 11836170001, Roche) with three 1-min pulses of bead-beating and 1 min of cooling on ice between each pulse. Note that detergent was omitted during homogenization to minimize foaming but was added for the solubilization of membrane proteins during the subsequent protein extraction. Specifically, homogenates were extracted with 1% NP40, 100 mM Tris–HCl, pH 8.3, 100 mM NaCl, 1 × Roche PhosSTOP phosphatase inhibitor cocktail, 1 × Roche cOmplete protease inhibitor cocktail, at 4 °C for 30 min with agitation before centrifugation at 4 °C for 5 min at 2000×*g* and 15 min at 21,130×*g*. Supernatants of brain protein extracts were collected following centrifugation, then protein concentrations were determined by bicinchoninic acid assay (catalog number 23227, Thermo Fisher Scientific) and equalized by dilution in extraction buffer. After samples were prepared with NuPAGE LDS Sample Buffer (catalog number NP0007, Thermo Fisher Scientific) containing 2% β-mercaptoethanol, and heated at 70 °C for 10 min, 30 to 60 µg of protein per lane were loaded and separated on 10% Bis–tris BOLT SDS-Page gels (catalog number NW00105BOX, Thermo Fisher Scientific) at 100 V for 1.5 h in MOPS buffer. Proteins were then transferred to 0.45 µm pore PVDF (catalog number IPVH00010, Millipore) at 25 to 35 V for 1 h and incubated with appropriate antibodies overnight at 4 °C, followed by 1-h incubation with Horseradish-Peroxidase-linked secondary antibodies at room temperature. Horseradish peroxidase-catalyzed chemiluminescence from SuperSignal West Dura Extended Duration HRP Substrate (catalog number 37071, Thermo Fisher Scientific) was detected on radiography film and band intensities were quantified on ImageJ software version 1.53e.

### Neprilysin activity measurements

To assess neprilysin activity, brain samples were analyzed using the Sensolyte 520 Neprilysin Activity Assay Fluorometric Kit (catalog number AS-72223, AnaSpec) and following the manufacturer’s instructions. Briefly, 10% brain extracts were generated and cleared of insoluble material as described in the western blot analysis section. Throughout these steps, the brain extracts were kept on ice. Next, 50 µL of brain extract supernatants were pipetted onto a 96-well black/clear bottom plate (catalog number 165305, Thermo Fisher Scientific) and 50 µL of 2 × Neprilysin Substrate (provided in the kit) was added to the samples for an assay concentration of 5 µM substrate. The 96-well plate was then placed in a 37 °C incubator for 1 h followed by fluorometric analysis using a SpectraMax i3x Multi-Mode Microplate Reader (Molecular Devices, San Jose, CA, USA) at 490 nm/520 nm excitation/emission wavelengths. As positive controls and to calibrate the system served recombinant neprilysin enzyme (provided in the kit and diluted to assay concentrations of 1 to 10 ng per well). Background activities toward the Neprilysin Substrate were determined by undertaking assay reactions in the presence of 100 nM levels of the neprilysin inhibitor thiorphan (provided in the kit). All analyses were undertaken in triplicate.

### Immunohistochemistry

Brain hemispheres were paraffin-embedded then sectioned parasagittally at 5-micron thickness, and three contiguous sections per animal from near lateral 0.1 mm were mounted to each slide. Tissue processing was based on the following steps: xylene deparaffinization, hydration in graded ethanol/water, peroxidase inactivation in methanolic 3% H_2_O_2_ (v/v) for 25 min, antigen retrieval in formic acid for 5 min, blocking with horse serum (catalog number MP-7802-15, Vector Laboratories, Burlingame, CA, USA), overnight 4G8 (1:3000) antibody application in mouse-on-mouse diluent (catalog number BMK-2202, Vector Laboratories). Staining of 4G8 immunoreactivity was with 3,3′-diaminobenzidine. Nuclei were stained with Harris hematoxylin (Sigma-Aldrich) then hydrochloric acid was used to differentiate the tissue.

Prepared slides were scanned at 20 × magnification (0.5 µm/pixel) on an Aperio Scanscope AT2 (Leica, Wetzlar, Germany) and plaque quantification was performed on HALO software (Version 2.3.2089.29, Indica labs, Albuquerque, NM, USA). Margins of the cortical and hippocampal regions were manually defined (as exemplified in Fig. 3B), creating two annotation layers per slide. The anterior olfactory nucleus and corpus callosum were included in the cortical annotation. Vasculature and meninges were manually excluded from the annotations. Classifiers for plaques (4G8-positive tissue), non-plaque (4G8-negative tissue) and background (voids in the tissue) were defined manually and iteratively to compensate for differences in background staining such that the minimum number of classifiers were applied to the dataset. Following automated detection of the classifiers, the quantified annotations of each slide were manually examined to verify that the classifier settings discriminated plaque, non-plaque, and background. Raw data (object data) from each analysis were exported in text format. The areas of all objects classified as plaques and non-plaques from a given annotation on a given slide were summed to give the total cortical or hippocampal area, respectively.

### Reverse transcription real-time quantitative polymerase chain reaction (RT-qPCR)

Working surfaces were treated with RNase Zap (catalog number AM9780, Thermo Fisher Scientific). Frozen left cerebral hemispheres were weighed then homogenized in QIAzol lysis reagent (catalog number 79306, Qiagen, Venlo, Netherlands) on ice with an immersion style tissue homogenizer for 40 s, then RNA was extracted and purified with an RNeasy kit (catalog number 74004, Qiagen) according to the manufacturer’s protocol. RNA integrity and yield were assessed by 1.5% agarose gel electrophoresis and UV–Vis spectroscopy (NanoDrop, Thermo Fisher Scientific), respectively. An AffinityScript cDNA synthesis kit (catalog number 200436, Agilent, Santa Clara, CA, USA) was used according to the manufacturer’s instructions to prepare cDNA libraries.

TaqMan™ Universal Master Mix II and FAM™-minor groove binder-labeled TaqMan probes (Cort: Mm00432631_m1, Mme: Mm00485028_m1, Ppia: Mm02342430_g1, Sst: Mm00436671_m1, Thermo Fisher Scientific) were mixed with cDNA in the manufacturer’s recommended volumetric ratios. A total of 180 ng of cDNA was used in each reaction to quantify all transcripts except *Mme*, for which 50 ng was used. Fluorescence curves were obtained on a Light Cycler 480 II thermal cycler (Roche) in runs starting with a 2-min 50 °C hold, then a 10-min 95 °C hold, then 40 cycles from 95 °C for 15 s to 60 °C for 1 min when fluorescence was measured at 465–510 nm. For each *App*^*NL-F/NL-F*^ genotype and each transcript studied, analyses were based on a minimum of three biological replicates. Quantification cycles (C_q_), also known as threshold cycles (C_t_), were determined using the second derivative maximum method. Sample-matched C_q_ values generated by the concomitant analysis of *Ppia* on each plate were used as references to calculate relative transcript abundances based on the delta-delta C_t_ method^38^.

### Neprilysin activity assay

To assess neprilysin activity in the brain extracts of 15-month-old *App*^*NL-F/NL-F*^ used for immunoblotting analysis, samples were analyzed using the Sensolyte 520 Neprilysin Activity Assay Fluorometric Kit (catalog number AS-72223, AnaSpec, Fremont, CA, USA) that employs a 5-FAM/QXL FRET substrate. Upon incubation with a sample of interest comprising neprilysin activity the 5-FAM component is freed from the substrate and can be quantified fluorometrically. Briefly, the 10% (w/v) brain extracts were centrifuged at 15,000 rcf for 5 min to sediment insoluble debris. 50 µL aliquots of the centrifuge supernatants were then pipetted into the wells of a 96-well black, flat-bottom plate (catalog number 165305, Thermo Fisher Scientific) along with 50 µL of 2 × 5-FAM/QXL FRET substrate. A total of four brain extracts each from wildtype and *Sst* knockout *App*^*NL-F/NL-F*^ mice were analyzed, with each sample being tested in three technical replicates. As a positive control served the FAM/QXL FRET substrate incubated with 10 ug/mL recombinant neprilysin provided in the kit. For the generation of negative background fluorescence controls, the substrate was replaced with water in the assay mix. The 96-well plate was then placed in a 37 °C incubator for 1 h followed by fluorometric analysis using a SpectraMax i3x Multi-Mode Microplate Reader at 490 nm excitation and 520 nm emission wavelengths and the activity was determined in relative fluorescence units, normalized to the recombinant neprilysin samples.

### Statistics

All processing of raw data and all statistical tests were undertaken with Microsoft Excel and GraphPad. This included RT-qPCR analyses collected with LightCycler 480 (version 1.5.1.62), immunohistochemistry data obtained with HALO software (Indica Labs, Albuquerque, New Mexico, USA), western blots scanned for densitometry analyses with ImageJ (National Institutes of Health and the Laboratory for Optical and Computational Instrumentation, University of Wisconsin, WI, USA), and neprilysin activity analyses. Specifically, the Microsoft Excel Analysis ToolPack and the GraphPad *t*-test calculator were used to compute *p-*values. In instances when more than two groups of samples were compared, we initially tested if the null hypothesis is valid using ANOVA, followed by pairwise t-tests and Bonferroni correction. All *t*-test were two-tailed based on the assumption that the variance of groups of samples was not known. Amyloid plaque image analysis data from cohorts of age- and Sst genotype-matched mice were assessed for normality using kurtosis and skewness values determined with the descriptive statistics function, where acceptable values for either metric were between − 2 and 2. Plaque density distributions were considered discrete. *p*-values are either shown as numbers or with asterisks according to conventions in the field, i.e., *p*-values lower than 0.05 were considered significant and were marked with a single asterisk. Additional asterisks are shown for every tenfold lower *p*-value. When *t*-tests failed to meet the significance threshold, the abbreviation ‘ns’ is shown.

### Ethics statement

All mouse handling procedures and experimental protocols were approved by the University Health Network Animal Care Committee (ACC) overseeing work at the Krembil Discovery Centre Animal Resource Centre (ARC) under Animal Use Protocol 4183. All methods were carried out in accordance with Animal Research: Reporting of In Vivo Experiments (ARRIVE) guidelines and requirements of the Canadian Council on Animal Care (CCAC). The use of mouse brains for biochemical applications detailed in this report was reviewed and authorized (Biosafety Permit 208-S06-2) by the Environmental Health a Safety (EHS) office at the University of Toronto, Toronto, Ontario, Canada.

## Results

### Generation of ***App***^***NL-F/NL-F***^***Sst***^***−/−***^ mouse line

**Figure 1:**
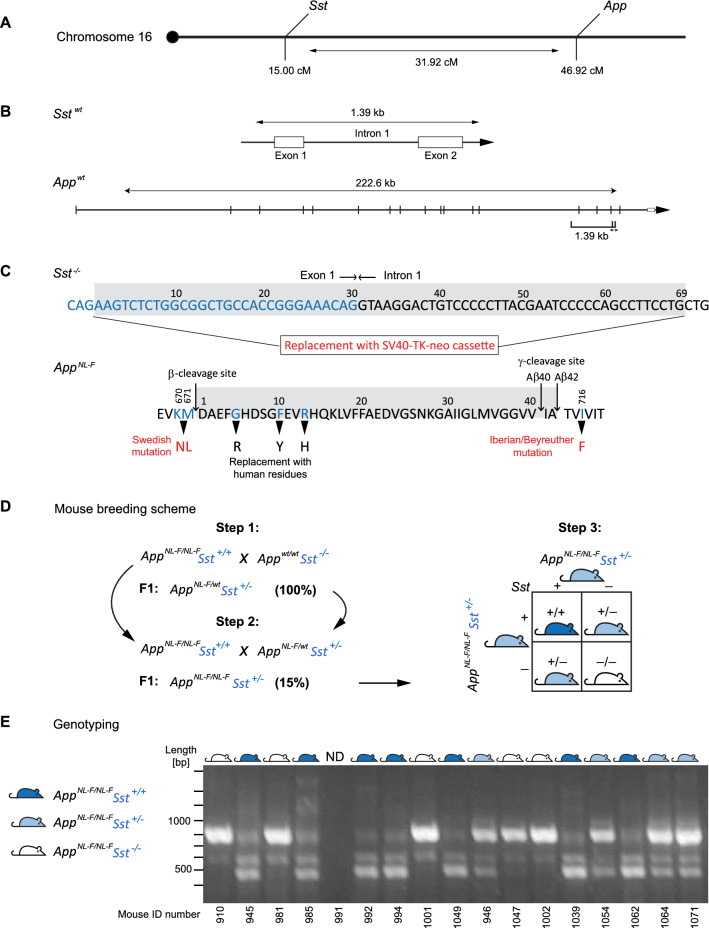
Generation of the *App*^*NL-F/NL-F*^*Sst*^*−/−*^ mouse line. (**A**) Depiction of the relative positioning of *Sst* and *App* genes on mouse Chromosome 16. (**B**) Intron–exon gene organizations of mouse *Sst* and *App* genes. (**C**) Summary of genomic alterations present in the *Sst*^*-/-*^ and *App*^*NL-F/NL-F*^ parental mouse lines used in this study. (**D**) Depiction of the three-step breeding scheme, including Punnett Square diagram of final intercrosses. (**E**) Representative results from tail clip-based genotyping analyses of *App*^*NL-F/NL-F*^ X *Sst*^+*/−*^ intercrosses.

Both the *Sst* and the *App* genes are coded on Chromosome 16 in mice, which could jeopardize the ability to generate mice carrying both the *App* gene mutation and *Sst* deletion through intercrossing if the genes were linked and recombination disfavored. A closer analysis mostly dispelled this concern because the *Sst* gene maps to cytoband qB1 (23889573–23890958 bp, Genome Reference Consortium mouse build 38/mm10) and the *App* gene is coded in cytoband qC3.3 (84952666–85175255 bp), located 32 cM apart (Fig. 1A).

Sst and App proteins are coded by multi-exon gene sequences (Fig. 1B). Several mice in which the *Sst* gene or the expression of Sst receptors were deleted have been reported in the literature^39^. Here we employed a well-validated *Sst* null line that was originally generated by the insertion of a SV40-TK-neo cassette, which had caused the deletion of 69 base pairs within the Exon 1-Intron 1 junctional sequence region within the *Sst* gene^37^ (Fig. 1C). To our knowledge the sequence of this insert was not originally published, which is why we inserted it here as a supplement (Supplementary Fig. 1). This *Sst* null line was selected because it had been reported to feature normal hippocampal App expression. Moreover, the aforementioned data, which suggested Sst-deficiency leads to diminished neprilysin activity and elevated Aβ_42_ levels, were also generated with this line^26^.

The selection of a compatible Aβ amyloidosis model fell on a previously reported knock-in mouse line sharing with the *Sst* null mice the same C57BL/6 inbred genomic background^36^. This *App*^*NL-F/NL-F*^ line carries several mutations that flank or alter the Aβ42 coding sequence. Cumulatively these changes humanize the endogenous mouse Aβ sequence, and were expected to increase, relative to wild-type mice, the production of Aβ and the ratio of Aβ42-to-Aβ40^36^. We chose this knock-in model to avoid insertion artefacts and variances that are frequently observed in transgenic lines due to the large and fluctuating number of transgene copies. Prior global proteome analyses of whole brain and hippocampus samples from these mice indicated that Sst expression is comparable in *APP*^*NL-F/NL-F*^ and APP^wt/wt^ control mice (Cort was not detected in these analyses)^40,41^.

To generate cohorts for this study, three consecutive intercrosses were undertaken. More specifically, we initially crossbred the *Sst* null line with homozygous *App*^*NL-F/NL-F*^ mice (Fig. 1D). Next, the F1 generation (*App*^*NL-F/wt*^*Sst*^+*/−*^) was backcrossed with *APP*^*NL-F/NL-F*^ mice to produce intercrosses that were homozygous for the *NL-F/NL-F* mutation but heterozygous for the Sst knockout (*App*^*NL-F/NL-F*^*Sst*^+*/−*^). Due to the need for a Chromosome 16 crossover event to achieve this genotype only approximately 15% of the F1 offspring from this second intercrossing, i.e., 13 out of a total of 87 mice, were confirmed as *App*^*NL-F/NL-F*^*Sst*^+*/−*^. Finally, *App*^*NL-F/NL-F*^*Sst*^+*/−*^ were intercrossed and progeny were genotyped from tail clippings using a customized PCR analysis (Fig. 1E). This scheme minimized undesired variability by ensuring that *Sst* null mice (*App*^*NL-F/NL-F*^*Sst*^*−/−*^) shared the same parents and housing as their wild-type *Sst* littermates (*App*^*NL-F/NL-F*^*Sst*^+*/*+^). Genotyping validated that *App*^*NL-F/NL-F*^*Sst*^*−/−*^, *App*^*NL-F/NL-F*^*Sst*^+*/−*^, and *App*^*NL-F/NL-F*^*Sst*^+*/*+^ progeny were obtained in approximate Mendelian ratios. *App*^*NL-F/NL-F*^*Sst*^+*/−*^ hemizygotes and *App*^*NL-F/NL-F*^*Sst*^*−/−*^ knockouts were physically and behaviorally unremarkable and lived past 16 months of age with a mortality equivalent to their *Sst* wild-type littermates.

### *Sst*,* Mme*, and* Cort *transcript expression in *App*^*NL-F/NL-F*^*Sst*^*−/−*^ mouse brains

**Figure 2:**
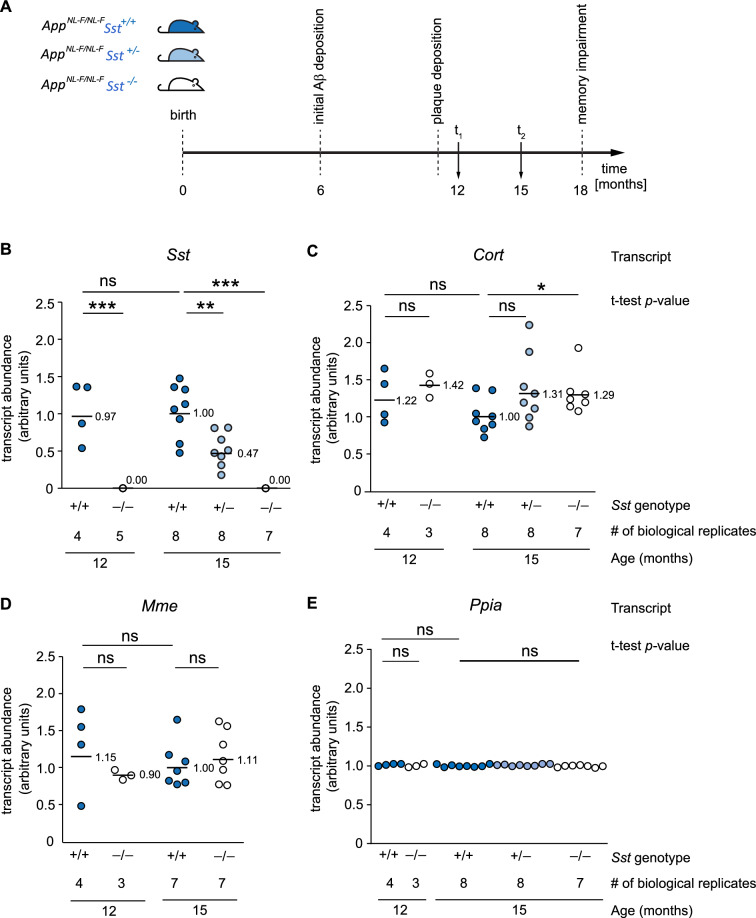
*Sst*-deficient *App*^*NL-F/NL-F*^*Sst*^*−/−*^ mice exhibit increased cortistatin and unaltered neprilysin transcript levels relative to *App*^*NL-F/NL-F*^*Sst*^+*/*+^ mice. (**A**) Timeline of relevant pathological events and ages selected for comparative RT-qPCR and neuropathological analyses. At times t_1_ and t_2_, mouse brains were bisected in the mid-sagittal plane. One half provided source material for RT-qPCR and western blot analyses, the other half was fixed in formalin and assessed for Aβ deposition by immunohistochemistry. (**B**) *Sst* transcript levels in whole brain extracts of *App*^*NL-F/NL-F*^*Sst*^+*/*+^, *App*^*NL-F/NL-F*^*Sst*^+*/−*^, and *App*^*NL-F/NL-F*^*Sst*^*−/−*^ mice. *Sst* transcript levels were lower in 15-month-old hemizygotes than in wild-types (p = 0.004, paired two-tailed t-test and Bonferroni correction) and were undetectable in 12- or 15-month-old *Sst* knockouts. (**C**) Brain *Cort* transcript levels did not differ significantly between *Sst* wild-type and hemizygous mice (paired two tailed t-test and Bonferroni correction) but were significantly increased in 15-month-old *Sst* knockout *App*^*NL-F/NL-F*^*Sst*^*−/−*^ mice, relative to *Sst* wild-type *App*^*NL-F/NL- F*^*Sst*^+*/*+^ mice. (**D**) Whole brain neprilysin (*Mme*) transcript levels were not affected by mono- or bi-allelic *Sst* knockout in *App*^*NL-F/NL-F*^*Sst*^*−/−*^ mice. (E) Relative *Ppia* mRNA levels in the brains of 12- and 15-month-old *App*^*NL-F/NL-F*^*Sst*^+*/*+^, *App*^*NL-F/NL-F*^*Sst*^+*/−*^, and *App*^*NL-F/NL-F*^*Sst*^*−/−*^ mice. Each dot is the value from one mouse. *Ppia* transcription was assumed to be constant in all *Sst* genotypes. All amplifications shown in subpanels of this figure were based on 180 ng cDNA. Bars are averages. ns, non-significant.

Prior work indicated that mRNA transcript levels of the *Sst* paralog *Cort* may undergo a gender-dependent compensatory increase in *Sst* knockout mice^42^. Moreover, transcription of the neprilysin gene (*Mme*) has been reported to be induced by Sst^26^, a finding that could have direct implications for interpreting effects of Sst on Aβ amyloid deposition, due to the enzyme–substrate relationship between neprilysin and Aβ. To explore these possibilities, we aged *Sst* wild-type, hemizygous, or knockout *App*^*NL-F/NL-F*^ mice to 12 or 15 months, ages at which the *App*^*NL-F/NL-F*^ genotype gives rise to subtle and prominent Aβ amyloid deposition respectively (Fig. 2A). We then determined transcript levels of *Sst*, *Mme*, *Cort*, and *Ppia*, the latter a common proxy for total RNA in RT-qPCR of brain tissue. As biological source materials served mid-sagittal cut half brains, as opposed to specific brain regions, to minimize variances that can be introduced during dissection steps.

Distributions of transcript abundance for *Sst* (Fig. 2B), *Cort *(Fig. 2C), and *Mme* (Fig. 2D) were approximately normal, and coefficients of variation ranged from 0.35 to 0.43, 0.12 to 0.34, and 0.07 to 0.44, respectively. Moreover, for all transcripts measured, mRNA levels in males and females were equivalent, as assessed by *t*-test. As expected, these analyses revealed that *Ppia* transcript levels were highly consistent between all samples (Fig. 2E), indicating that both the neurophysiological backgrounds sampled, and the sample preparations were consistent.

The brain *Sst* mRNA concentration of *App*^*NL-F/NL-F*^*Sst*^+*/−*^ hemizygotes averaged around half that of *Sst* wild-type *App*^*NL-F/NL-F*^*Sst*^+*/*+^ animals, suggesting that in most hemizygote animals a compensatory transcriptional upregulation of the remaining *Sst* allele did not occur (Fig. 2B). However, overlapping distributions of *Sst* transcript levels in *Sst* wild-type *App*^*NL-F/NL-F*^*Sst*^+*/*+^ and *Sst* hemizygote *App*^*NL-F/NL-F*^*Sst*^+*/−*^ mice indicated a degree of flexibility of *Sst* expression and raised the possibility that some hemizygous individuals may produce sufficient levels of *Sst* to be phenotypically wild-type. The *Sst* transcript was undetectable in all *App*^*NL-F/NL-F*^*Sst*^*−/−*^ mice, confirming that the mutated allele was not expressed.

At both ages examined, *Sst* wild-type *App*^*NL-F/NL-F*^*Sst*^+*/*+^ mice had consistent levels of *Cort*, *Mme*, and *Sst* transcripts, indicating that each of these genes was stably expressed over the 3 month age range (Fig. 2B–D). *Cort* mRNA transcript levels increased in *Sst*-deficient *App*^*NL-F/NL-F*^*Sst*^*−/−*^ mice by approximately one third (35%), relative to age-matched *Sst* wild-type *App*^*NL-F/NL-F*^*Sst*^+*/*+^ mice. This increase was significant (*p* < 0.05) between *Sst* wild-type and knockout *App*^*NL-F/NL-F*^ cohorts at 15 months of age (Fig. 2C). Remarkably, there was no significant difference in neprilysin transcript levels between *Sst* wild-type *App*^*NL-F/NL-F*^*Sst*^+*/*+^ and *Sst* knockout *App*^*NL-F/NL-F*^*Sst*^*−/−*^ animals at 12 or 15 months of age (Fig. 2D).

In summary, these RT-qPCR data validated a compensatory increase in *Cort* mRNA levels in response to *Sst* knockout but did not corroborate the previously reported male sex-specificity of this increase^42^. These data also did not validate the concept of Sst acting as an inducer of whole brain neprilysin expression.

### Amyloid plaque deposition in *App*^*NL-F/NL-F*^*Sst*^*−/−*^ mice

**Figure 3:**
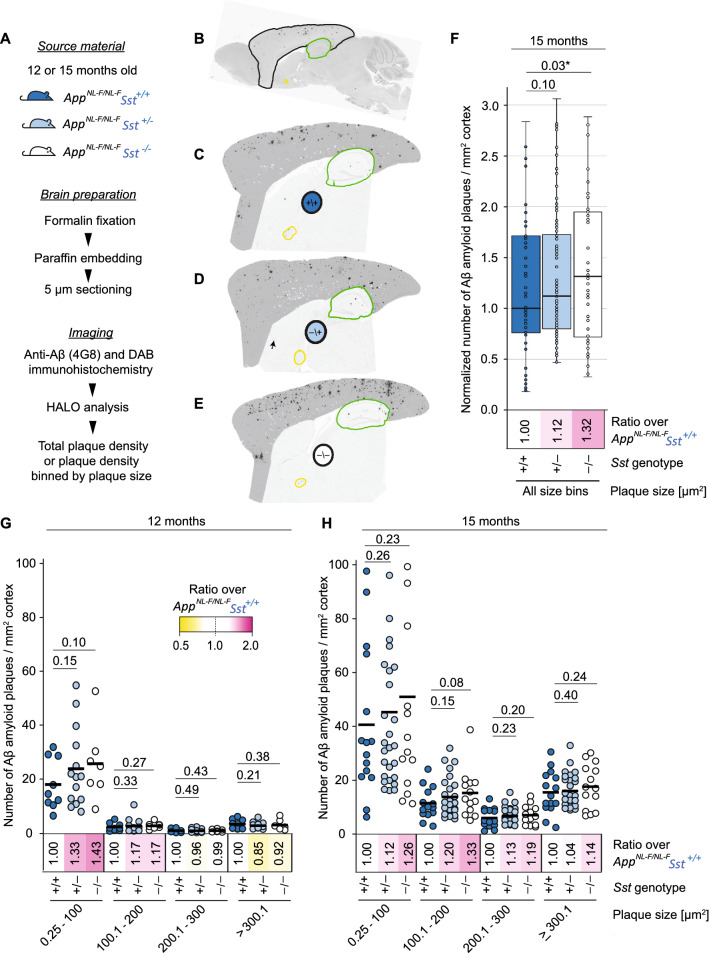
Cortical Aβ plaque density is increased in *App*^*NL-F/NL-F*^ mice upon *Sst* ablation. (**A**) Workflow for the assessment of Aβ plaque distribution and density. (**B**) Parasagittal brain section highlighting cortical and hippocampal areas that were separately analyzed for plaque density and are shown bounded in black and green, respectively. The anterior commissure is outlined in yellow. The images were generated with HALO software (Version 2.3.2089.29; https://indicalab.com/halo/) and margins of subregions were manually defined. Representative micrographs of parasagittal brain sections from 15-month-old (**C**) *App*^*NL-F/NL-F*^*Sst*^+*/*+^, (**D**) *App*^*NL-F/NL-F*^*Sst*^+*/−*^, and (**E**) *App*^*NL-F/NL-F*^*Sst*^*−/−*^ mice stained with 4G8-DAB showing plaques are largely restricted to cortical and hippocampal areas. (**F**) Plaque density measurements were based on all plaques exceeding 0.25 µm and were normalized relative to the median plaque density observed in *App*^*NL-F/NL-F*^*Sst*^+*/*+^ cortices. Paired two-tailed t-test results indicate a significant relative increase in plaque density in *App*^*NL-F/NL-F*^*Sst*^*−/−*^ cortices relative to side-by-side processed cortices derived from age-matched *App*^*NL-F/NL-F*^*Sst*^+*/*+^ mice. (**G**) Comparison of cortical Aβ amyloid plaque densities, binned into four Aβ amyloid plaque size ranges, in 12-month and (**H**) 15-month old *App*^*NL-F/NL-F*^ mice with wild-type, hemizygous, or knockout *Sst* genotype. Aβ plaque densities increased in all four plaque size categories in the older animals, irrespective of *Sst* genotype. A consistent trend, indicating an increase in plaque density, yet not meeting significance conventions (*p* < 0.05), was observed in all four plaque size ranges in 15-month-old mice, when *Sst* hemizygous or deficient mice were compared against wild-type *Sst* expressing *App*^*NL-F/NL-F*^ mice. Bonferroni-corrected *p-*values are shown in the graphs.

The RT-qPCR data may lead to an anticipation that the compensatory increase in *Cort* transcript levels could diminish effects of *Sst* knockout on Aβ deposition. To address this question experimentally, we next assessed cohorts of *App*^*NL-F/NL-F*^Sst^+/+^, *App*^*NL-F/NL-F*^*Sst*^+*/−*^ and *App*^*NL-F/NL-F*^*Sst*^*−/−*^ mice for Aβ deposition at 12 and 15 months of age. To this end, half brains were formalin fixed, paraffin-embedded and parasagittally cut. Next, 4G8 anti-Aβ immunoreactivity was visualized with the horseradish peroxidase substrate 3, 3-diaminobenzidine, and Aβ plaque densities were quantified (Fig. 3A). Immunoreactivity was confined mainly to the cortex, hippocampus, and olfactory bulb, as evidenced in representative immunohistochemical sections shown (Fig. 3B–E). A small number of amyloid plaques were occasionally observed within the corpus callosum and the anterior olfactory nucleus, particularly in brain sections of 15-month-old mice. The brainstem, midbrain, and cerebellum were devoid of 4G8 immunoreactivity in all samples. Of all cortical areas, the prelimbic and orbital areas generally had the lowest plaque density. Cortical plaque density increased from 12 to 15 months of age in each *Sst* genotype (*App*^*NL-F/NL-F*^*Sst*^+*/*+^: 3.7 times; *App*^*NL-F/NL-F*^*Sst*^+*/−*^: 4.6 times; *App*^*NL-F/NL-F*^*Sst*^*−/−*^: 4.5 times). At both ages studied, the sex of the animals did not appear to influence the propensity or speed of plaque formation that we captured in these analyses.

When comparing the cortical Aβ amyloid plaque density for all plaques larger than 0.25 µM in diameter (the smallest plaque size that could be reliably detected in these analyses), we observed that *Sst*-deficient *App*^*NL-F/NL-F*^*Sst*^*−/−*^ mice exhibited a statistically significant increase in plaque density, relative to *Sst* wild-type *App*^*NL-F/NL-F*^*Sst*^+*/*+^ at 15 months (Fig. 3F).

To dig deeper into how *Sst* deficiency might affect Aβ amyloid deposition, we segregated Aβ plaques with diameters up to 300 µM into three equally proportioned size ranges. Perhaps not surprisingly, the smallest plaque size range contained the largest proportion of total plaques in all three mouse populations at both ages. We were particularly interested in the relative densities of amyloid plaques of the smallest size in 12-month-old mice, hypothesizing that *Sst* influences the earliest steps of Aβ aggregation. The *Sst*-associated difference in plaque densities was indeed most pronounced at this relatively early timepoint in the size range that included the smallest detectable plaques (0.25–100 µm) but did not reach statistical significance (Fig. 3G). In the second smallest size range examined (100–200 µm), *Sst*-deficient mice had 17% more plaques than Sst wild-types. Larger plaques (> 200 µm) made up a smaller proportion of the total plaque area, namely 16%, 12%, and 11% of total plaques in *Sst* wild-type, hemizygous and deficient *App*^*NL-F/NL-F*^ mice, respectively, and appeared equally abundant in 12-month-old mice in all three *Sst* genotypes. At 15 months, *Sst* knockout *App*^*NL-F/NL-F*^*Sst*^*−/−*^ had consistently higher cortical plaque burdens than *Sst* wild-type mice across all plaque sizes, and hemizygote *App*^*NL-F/NL-F*^*Sst*^+*/−*^ animals presented with intermediate plaque burdens (Fig. 3H). Statistical analyses confirmed these trends yet did not establish significance for any of the pair-wise comparisons.

Hippocampal amyloid plaque density in 12- and 15-month-old *App*^*NL-F/NL-F*^ mice of all three *Sst* genotypes was considerably lower than that of the cortical regions. As in the cortex, hippocampal plaques up to 200 µm were more abundant in 12-month-old Sst-deficient mice and this trend extended to all size categories at 15 months of age (Supplementary Fig. 2).

Taken together, these analyses corroborated the notion that the presence of a functional *Sst* gene slows the formation of Aβ amyloid plaque deposition in *APP*^*NL-F/NL-F*^ mice.

### Steady-state protein levels and activity of neprilysin in mouse brain extracts

**Figure 4:**
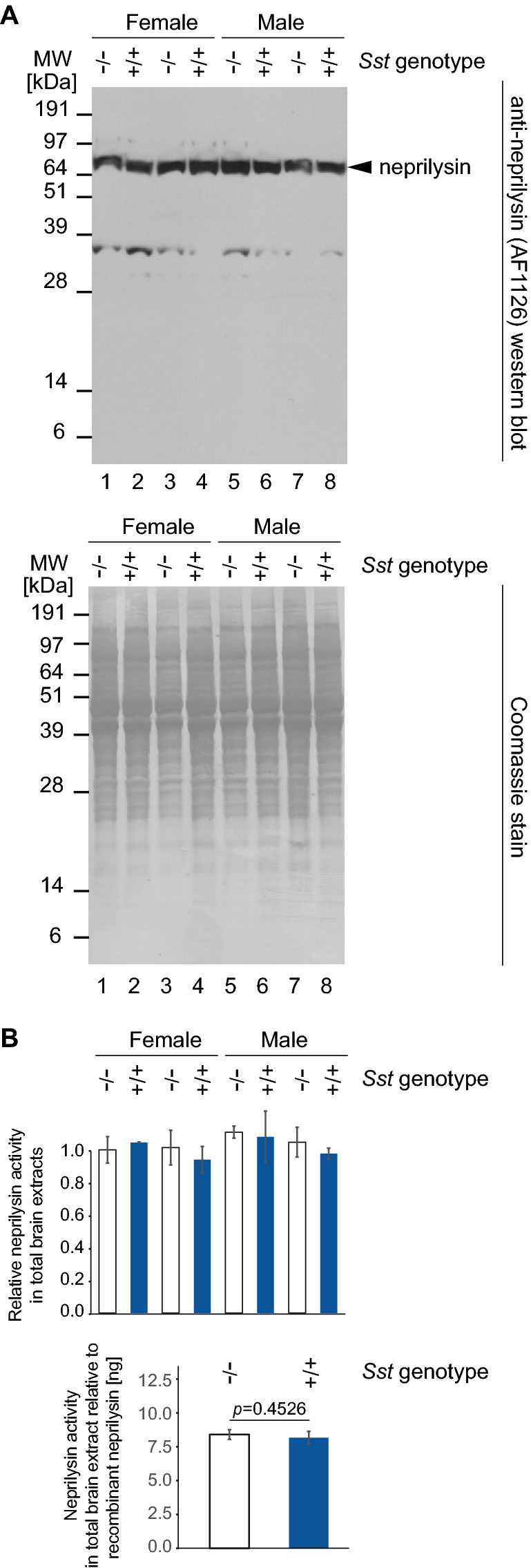
No detectable change in steady-state neprilysin protein levels or activity in Sst-deficient *App*^*NL-F/NL-F*^ mice. (**A**) *Sst* gene knockout had no effect on steady-state neprilysin protein levels in extracts from mid-sagittal cut half brains of *App*^*NL-F/NL-F*^ mice. The lower panel depicts a Coomassie stain of the SDS-PAGE-separated brain extract fractions used in these analyses whose total protein levels had been adjusted by bicinchoninic acid analysis. (**B**) *Sst* gene knockout had no significant effect on neprilysin activity present in brain extracts from mid-sagittal cut half brains of *App*^*NL-F/NL-F*^ mice toward an internally quenched 5-carboxyfluorescein (5-FAM) conjugated neprilysin substrate. Whereas the upper chart shows the neprilysin activities within Sst wild-type and deficient *App*^*NL-F/NL-F*^ brain extracts relative to each other (normalized to the mean neprilysin activity measured in one of the Sst-deficient *App*^*NL-F/NL-F*^ mice), the lower chart depicts their mean neprilysin activities relative to the activity of a known amount of recombinant human neprilysin. Original western blots and their Coomassie-stained images are presented in Supplementary Fig. 3.

Although we did not observe Sst-dependent differences in neprilysin transcript levels, we wondered if Sst increases neprilysin steady-state protein levels. To investigate this possibility, we quantified neprilysin protein levels in NP-40 solubilized brain extracts (derived from mid-sagittal cut half brains) from 15-month-old mice by western blot analyses. Analogous to the RT-qPCR results, this experiment revealed consistent steady-state neprilysin levels and no apparent changes in post-translational modifications between *Sst* wild-type *App*^*NL-F/NL-F*^*Sst*^+*/*+^ and knockout *App*^*NL-F/NL-F*^*Sst*^*−/−*^ mice (Fig. 4A). Next, we measured the in vitro activity of *Sst* wild-type and deficient *App*^*NL-F/NL-F*^ brain extracts toward a commercial fluorescent neprilysin substrate (5-FAM/QXL-520). In these analyses, background (non-neprilysin) activities within brain extracts toward the fluorescent substrate were elucidated by incubating brain extracts in the presence of the neprilysin-specific inhibitor thiorpan (Fig. 4B). To estimate total neprilysin activity levels in our extract fractions we compared their activity to the activity of a known amount of recombinant human neprilysin. These analyses established that the presence or absence of Sst did not affect neprilysin expression or activity in a significant manner in total brain extracts.

### *Sst* ablation promotes the formation of high molecular mass Aβ oligomers

**Figure 5:**
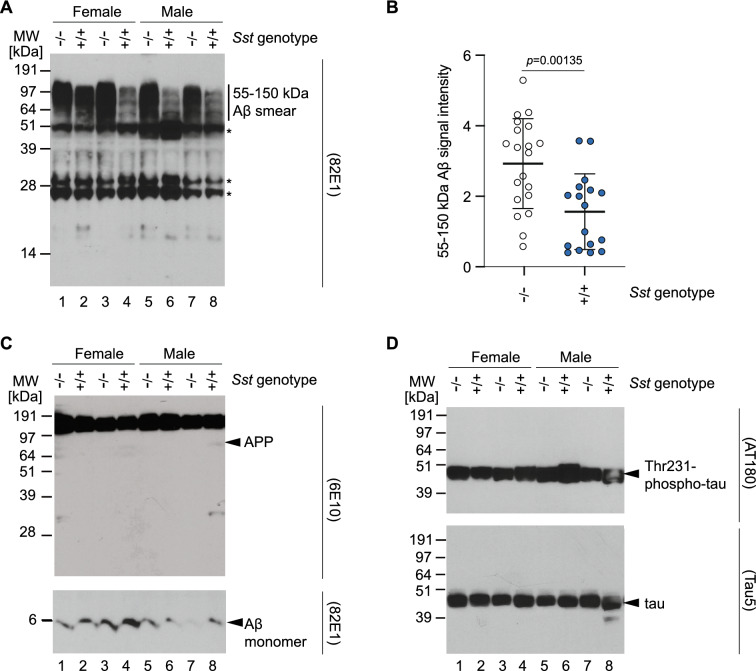
*Sst* ablation promotes the formation of high molecular mass Aβ oligomers. (**A**) *Sst* gene knockout is associated with an increase in the intensity of diffuse 82E1 signals observed at 55–150 kDa in western blot analyses of *App*^*NL-F/NL-F*^ mouse-derived brain extracts. Bands indicated in this panel with asterisks represent signals generated by the secondary anti-mouse antibody reacting with endogenous IgG present in the mouse brain extracts. IgG was identified based on bands of the same size and relative intensity also appearing when the same anti-mouse secondary antibody was applied without primary antibody to brain extract fractions. (**B**) ImageJ-based densitometry analyses of western blot bands shown in Panel A within the 55–150 kDa apparent molecular weight range validate that the increase in signal intensities is significant (*p* = 0.0052) when comparing *Sst* wild-type and deficient *App*^*NL-F/NL-F*^ mice. (**C**) Germline *Sst* ablation does not translate into differences in total APP or monomeric Aβ levels in 15-month-old *App*^*NL-F/NL-F*^ mice, detected with 6E10 and 82E1 (following long exposure) antibodies in western blots, respectively. (**D**) Although AT180 phospho-tau western blot signal intensities were subject to small fluctuations in mouse brain extracts of 15-month-old *App*^*NL-F/NL-F*^, these fluctuations did not track with the expression or loss of *Sst* gene expression. Similarly, no *Sst* gene associated differences in total tau western blot signal levels were observed in these 15-month-old *App*^*NL-F/NL-F*^ mice. Fractions analyzed in this figure were adjusted for total protein levels using the bicinchoninic acid method and were identical to those depicted following Coomassie staining in Fig. 4A. Original western blots are presented in Supplementary Fig. 3.

We considered that Sst may influence the earliest Aβ aggregation steps, namely oligomerization. This idea was based on our earlier observation that the addition of Sst to a synthetic Aβ_1–42_ preparation delays Aβ in vitro aggregation in a concentration-dependent manner^31^ and on data from molecular dynamics simulations by our collaborators showing that the presence of Sst interferes with early Aβ oligomer assembly^43^. We made use of the monoclonal 82E1 antibody, which exclusively detects Aβ assemblies with exposed Aβ N-termini and, consequently, will not bind APP or its derivatives encompassing the noncleaved Aβ sequence^44^. The same 15-month-old brain extracts analyzed by 82E1 western blot had striking differences between *Sst* wild-type *App*^*NL-F/NL-F*^*Sst*^+*/*+^ and knockout *App*^*NL-F/NL-F*^*Sst*^*−/−*^ mice: Whereas *Sst* knockout *App*^*NL-F/NL-F*^*Sst*^*−/−*^ mice gave a strong 82E1-reactive smear covering apparent molecular weights of 55–150 kDa, wild-type *App*^*NL-F/NL-F*^*Sst*^+*/*+^ gave rise to considerably weaker signals in this molecular weight range (Fig. 5A). Densitometry showed that the difference in signal intensities in this molecular weight range were highly significant (Fig. 5B).

Next, we generated western blots with the 6E10 anti-Aβ epitope, which predominantly detects full-length APP in extracts of 15-month-old *App*^*NL-F/NL-F*^ upon low exposure of blots, to determine if *Sst* influences the expression of this precursor. We also produced a long exposure of the low molecular weight region of the 82E1 western blot for the detection of monomeric Aβ (Fig. 5C). Finally, we probed western blots with antibodies directed against total tau and a prominent tau phosphorylation epitope encountered in AD brain samples (Fig. 5D). No changes in APP, monomeric Aβ, total tau or phospho-tau were observed.

Taken together, experiments in this section revealed that Sst slows Aβ deposition primarily through a neprilysin-independent effect on Aβ oligomerization that appears to affect early steps in Aβ oligomer assembly. Accordingly, Sst ablation increased the formation of SDS-resistant Aβ oligomers but did not influence levels of the APP precursor or the production of monomeric Aβ.

## Discussion

This study was designed to reveal whether *Sst* gene ablation affects Aβ amyloidosis in vivo. To this end, we crossed the extensively studied C57BL/6-derived *Sst* null mouse line^37^ and *App*^*NL-F/NL-F*^ mice^36^. The *Sst* gene knockout was validated by genotyping and RT-qPCR and was observed to cause a slight compensatory upregulation of the mRNA levels of the *Sst* paralog *Cort*. Remarkably, neither whole brain neprilysin transcript nor protein or activity levels were impacted by *Sst* genotype, yet *Sst* ablation still led to significantly stronger cortical Aβ deposition in 15-month-old *App*^*NL-F/NL-F*^ mice. When Aβ plaques were binned by size in 12-month-old *App*^*NL-F/NL-F*^ mice, which are at early stages of plaque deposition, it became apparent that the *Sst* gene ablation promoted the formation of the smallest Aβ plaques. A follow-on investigation into the mechanism revealed that in the absence of Sst the signal intensities of SDS-stable Aβ oligomers were strongly increased relative to wild-type *App*^*NL-F/NL-F*^ mouse brain levels, in the absence of an effect on APP precursor or monomeric Aβ levels. No *Sst* gene-associated effect on tau protein levels or phosphorylation were observed.

This study draws into question widely held concepts regarding the dominant mechanism by which Sst influences Aβ deposition in AD. For a more nuanced interpretation, it is critical to consider prior pertinent data. In 2005, a landmark study based on the same *Sst* knockout line employed in this work reported that neprilysin protein levels and activity were lower in the hippocampi, but not the cortices or cerebella, of *Sst* null mice with a corresponding increase in Aβ levels restricted to the hippocampus^26^. The authors proposed a model whereby the effect of the *Sst* gene knockout on Aβ levels is mediated by the previously established ability of neprilysin to digest Aβ; in other words, it was concluded that Sst acts indirectly on Aβ by inducing the expression of neprilysin. No data on Aβ aggregation were generated at the time because the mice employed only produce endogenous levels of mouse Aβ, and even a full knockout of the Aβ-degrading enzyme neprilysin does not generate Aβ amyloidosis in this model^45^. Nonetheless, the authors proposed that this phenomenon might also explain how diminishing SST levels, which have long been known to drop faster in late-onset AD cases relative to age-matched healthy elderly individuals^16,17,46,47^, may contribute to the disease. More recently, the main findings of this earlier study were corroborated, as repeated injections of Sst recombinantly fused to a blood brain barrier transporter (SST-scFv8D3) increased hippocampal neprilysin expression and reduced membrane-bound hippocampal Aβ_42_ in transgenic mice engineered to overexpress human APP harboring the Swedish mutation^48^. Although the use of an AD mouse model could have lent itself to observing whether SST modulated Aβ aggregation, this was not investigated.

The present work revealed whole brain neprilysin levels to be unaffected by *Sst* ablation at the mRNA or protein levels. It could be said therefore that a caveat in the interpretation of these divergent results is the difference in brain structures analyzed, i.e., hippocampus versus whole brain, including neprilysin-rich structures (basal ganglia, pituitary), which could dilute hippocampus-specific effects. Consistent with this interpretation, the difference in plaque numbers that we observed at 12 months were more pronounced in the hippocampus than cortical areas. More specifically, only in the hippocampi of 12-month-old mice did any plaque size range (plaques with areas 100.1–200 square micrometers) satisfy the t-test 0.05 confidence threshold. As we did not survey RNA expression in the hippocampus specifically, we cannot reject the hypothesis that this Sst-dependent local inhibition of plaque formation is due to a functional interaction between Sst and neprilysin. Future analyses of hippocampal neprilysin levels in *App*^*NL-F/NL-F*^*Sst*^+*/*+^ *and App*^*NL-F/NL-F*^*Sst*^*−/*−^ mice should clarify this issue. That said, according to recent Human Protein Atlas data (https://www.proteinatlas.org/ENSG00000196549-MME/brain) the hippocampus expresses a small fraction of total brain neprilysin mRNA and protein, such that if hippocampal neprilysin levels were to increase several-fold while remaining constant elsewhere, total expression would still be subtly affected^49^. Therefore, one conclusion of our study is that Sst is likely to play a lesser overall role for the expression of neprilysin throughout the brain than was previously appreciated.

We are not the first to suggest that the relationship between Sst and neprilysin expression may be limited to specific brain structures. For instance, the initial report establishing this relationship noted that in the cerebellum neprilysin is insensitive to *Sst* gene ablation^26^. More recently, a reduction in the levels of human SST and CORT in the temporal lobe of AD postmortem brains could not be correlated with neprilysin levels^17^. Finally, the aforementioned administration of the Sst-fusion construct only changed neprilysin levels in the hippocampus^48^.

**Figure 6:**
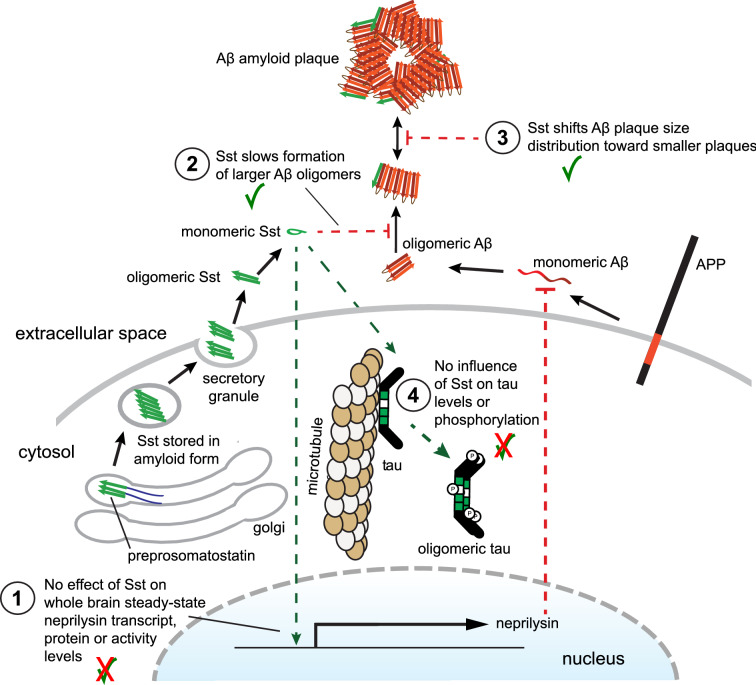
Cartoon summarizing key findings of this study. In 15-month-old *App*^*NL-F/NL-F*^ mice, the presence or absence of Sst has no effect on whole-brain steady-state neprilysin transcript, protein, or activity levels (1). Instead, Sst slows the formation of Aβ oligomers that can be visualized as 55–150 kDa SDS-resistant 82E1-reactive bands (2) and shifts Aβ amyloid formation and deposition (3). No effect of Sst was observed on steady-state tau protein levels or the tau phosphorylation at key AD phospho-acceptor sites. This cartoon was generated with Adobe Illustrator (version 5.9.0.372; https://www.adobe.com/). A similar cartoon was originally created and published under CC BY 4. 0 open access license by a subset of the authors (see Fig. 2 in^12^). Because these authors had retained copyright, no permission were needed for them to make the adjustments to the cartoon implemented here.

How else, if not based on SST controlling whole brain neprilysin expression, can we account for our observation that SST ablation increases the proportion of small plaques? We previously showed that Sst binds to oligomeric Aβ and modulates Aβ aggregation in vitro, preventing the formation of amyloid fibrils that can incorporate thioflavin T^31^. A closer look at fractions generated by co-incubation of Aβ and Sst showed that Sst delayed Aβ aggregation by stabilizing smaller oligomeric Aβ structures. More recently, a separate group reported that SST can bind certain Aβ oligomers^32^. Consistent with these earlier data, the in vivo immunohistochemical observations reported here support the notion that Sst inhibits early events in amyloid plaque formation such that plaques form more readily in *Sst* knockouts, then grow at a rate unaffected by *Sst* genotype (Fig. 6).

Arguably, our most instructive data for mechanistic modelling come from the western blotting of brain extracts derived from 15-month-old *App*^*NL-F/NL-F*^*Sst*^+*/*+^ *and App*^*NL-F/NL-F*^*Sst*^*−/*−^ mice. These western blot data established that the presence of Sst reduced the levels of 55–150 kDa bands that were reactive to an Aβ-specific antibody. That these signals originated from Aβ oligomers, as opposed to another APP isoform or cleavage product, can be deduced from the specificity of the antibody used, which is known to selectively target the N-terminal cleavage site of Aβ. Less obvious is whether the Sst-dependent slowing of Aβ aggregation would be therapeutic or detrimental for individuals with AD. Although it may be more intuitive to interpret the role of Sst as beneficial, whether this is true will depend on where exactly in the Aβ cascade Sst interferes, i.e., whether the presence of Sst increases or decreases the levels of the most toxic Aβ aggregation intermediates.

Could the *Sst* paralog *Cort* have compensated for the loss of *Sst* in this Aβ amyloidosis paradigm? This possibility is not far-fetched: Our prior in vitro data established that both Sst and Cort can interfere with Aβ aggregation, with Cort being the more potent inhibitor^31^. Specifically, we had previously shown that these peptides interfered with Aβ_1–42_ aggregation in a concentration-dependent manner when they were added to the seeding reaction at low micromolar concentrations and Aβ_1–42_ aggregation was monitored by ThT fluorescence. Moreover, a compensatory increase in hypothalamic *Cort* mRNA was documented in male *Sst* knockout mice^42^. Consistent with this previous report, we also found *Cort* transcript levels to increase in *Sst* null mice by around 35%. In contrast to the prior work, however, we observed no difference in this regard between male and female *App*^*NL-F/NL-F*^*Sst*^*−/−*^ mice. This apparent distinction may merely reflect our inclusion of the cerebrum, cerebellum, and caudoputamen, areas of the brain that express *Cort* and perhaps obscured sex-specific differences that may still exist in the hypothalamus.

When comparing female and male *APP*^*NL-F/NL-F*^ mice of each of the three *Sst* genotypes, we observed no sex-specific differences in cortical and hippocampal plaque density and plaque size distributions. In the absence of a sex-specific phenotype, which could have clarified the possible influence of a compensatory biology, our data cannot resolve the extent to which *Cort*-based compensation may have precluded the formation of more striking differences in AD-like Aβ amyloidosis. A *Cort* null mouse^50^ and an *Sst/Cort* double knockout mouse^51^ have been reported, raising the possibility that a triple transgenic line could be produced.

Another factor that could have masked the physiological influence of Sst on Aβ amyloidosis and AD molecular signatures in this study is the artificially high level of Aβ_1–42_ that the *App*^*NL-F/NL-F*^ knock-in mice are known to produce. This caveat represents a conceptual catch-22 because the very ability to measure the influence of Sst on Aβ amyloidosis depends on a paradigm that can produce sufficient Aβ levels to study its aggregation. Thus, any anticipation that the ablation of endogenous Sst would by itself profoundly affect amyloid plaque formation in *APP*^*NL-F/NL-F*^ mice needs to be tempered by the recognition that Aβ deposition in this model depends on Aβ_1–42_ production that far exceeds physiological Aβ production in human AD brains. When considered in this context, it is remarkable that *Sst* gene ablation alone was sufficient to increase the levels of SDS-stable Aβ assemblies to the extent seen.

## Conclusions

The results of this study document that Sst does not alter steady-state neprilysin transcript, protein or activity levels in whole brain extracts yet represent robust in vivo evidence of an inverse relationship between Sst levels and Aβ deposition. As such, the results are easier reconciled with a model of Sst affecting Aβ aggregation directly, consistent with recent biochemical and molecular dynamics simulation studies. This scenario also fits with results from new work documenting a relatively close spatial correlation of Sst and Aβ release sites in various brain regions^52^. Future work will need to establish if the combined ablation of the *Sst* and *Cort* genes will further enhance the impact on Aβ amyloid deposition. Once we understand if the Sst-mediated slowing of Aβ aggregation reduces or exacerbates toxicity in vivo, refined strategies should come to the fore that can harness the still untapped therapeutic potential of this cyclic neuropeptide for the treatment of AD.

## Supplementary Information

Supplementary Figures.

## Data Availability

All relevant data are available within the manuscript and its Supplementary Information files.

## Abbreviations

AD: Alzheimer’s disease
Aβ: Amyloid beta
APP: Amyloid precursor protein
Cort: Cortistatin
IDE: Insulin degrading enzyme
Mme: Neprilysin gene
Ppia: Peptidylprolyl isomerase A
Sst: Somatostatin
Sstr: Somatostatin receptor
