# Supplementary material for: Somatostatin slows Aβ plaque deposition in aged APPNL-F/NL-F mice by blocking Aβ aggregation
Source: Sci Rep. 2023 Feb 9;13:2337. doi: 10.1038/s41598-023-29559-z (PMC9911728; doi:10.1038/s41598-023-29559-z)

January 9, 2023

SUPPLEMENTARY INFORMATION

Somatostatin slows Aβ plaque deposition in aged *APP*$^{NL-F/NL-F}$ mice by blocking Aβ aggregation

Declan Williams[1], Bei Qi Yan[1,2], Hansen Wang[1], Logine Negm[1], Christopher Sackmann[1], Claire Verkuyl[1,2], Vanessa Rezai-Stevens[1], Shehab Eid[1,2], Nimit Vediya[1], Christine Sato[1], Joel C. Watts[1,3], Holger Wille[4,5], Gerold Schmitt-Ulms[1,2]*

[1] Tanz Centre for Research in Neurodegenerative Diseases

[2] Department of Laboratory Medicine & Pathobiology, University of Toronto

[3]Department of Biochemistry, University of Toronto, Toronto, Ontario, Canada.

[4]Department of Biochemistry, University of Alberta, Edmonton, Canada;

[5]Centre for Prions and Protein Folding Diseases, University of Alberta, Edmonton, Canada.

*Please address correspondence to: g.schmittulms@utoronto.ca

Supplementary Figure 1. Indel sequence of *Sst*$^{-/-}$ allele determined by Sanger sequencing.

In addition to depicting the indel, the image shows the position of forward and reverse primers used in this study for genotyping.

Supplementary Figure 2. Hippocampal Aβ amyloid plaque counts in 12- or 15-month-old *App*$^{NL-F/NL-F}$ mice that express wild-type Sst levels or were Sst-deficient.

Hippocampal Aβ amyloid plaque densities increased between 12- and 15-month-old *App*$^{NL-F/NL-F}$ mice. Differences in hippocampal Aβ amyloid plaque densities were observed when comparing *App*$^{NL-F/NL-F}$ mice that expressed wild-type Sst versus Sst-deficient mice. More specifically, a trend toward higher Aβ plaque densities of small sizes (0.25-200 µm) was observed in 12-month-old *Sst* gene-deficient mice, echoing the increase in Aβ amyloid plaque densities observed in the cortex of *Sst* ablated *App*$^{NL-F/NL-F}$ mice (**Fig. 3F**).

Supplementary Figure 3. Original images of Coomassie stains and western blots.

Supplementary Figure 1

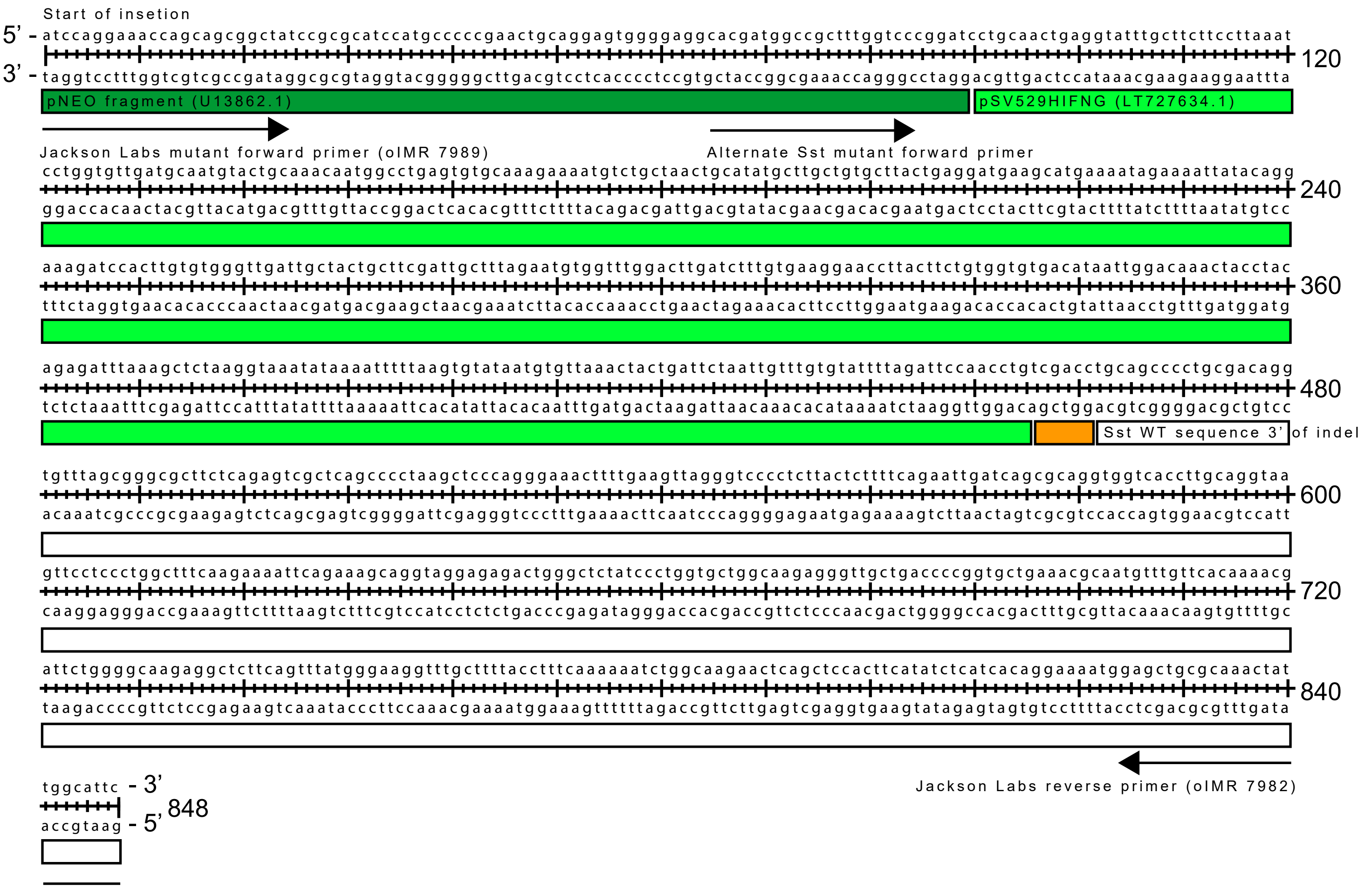

Supplementary Figure 2

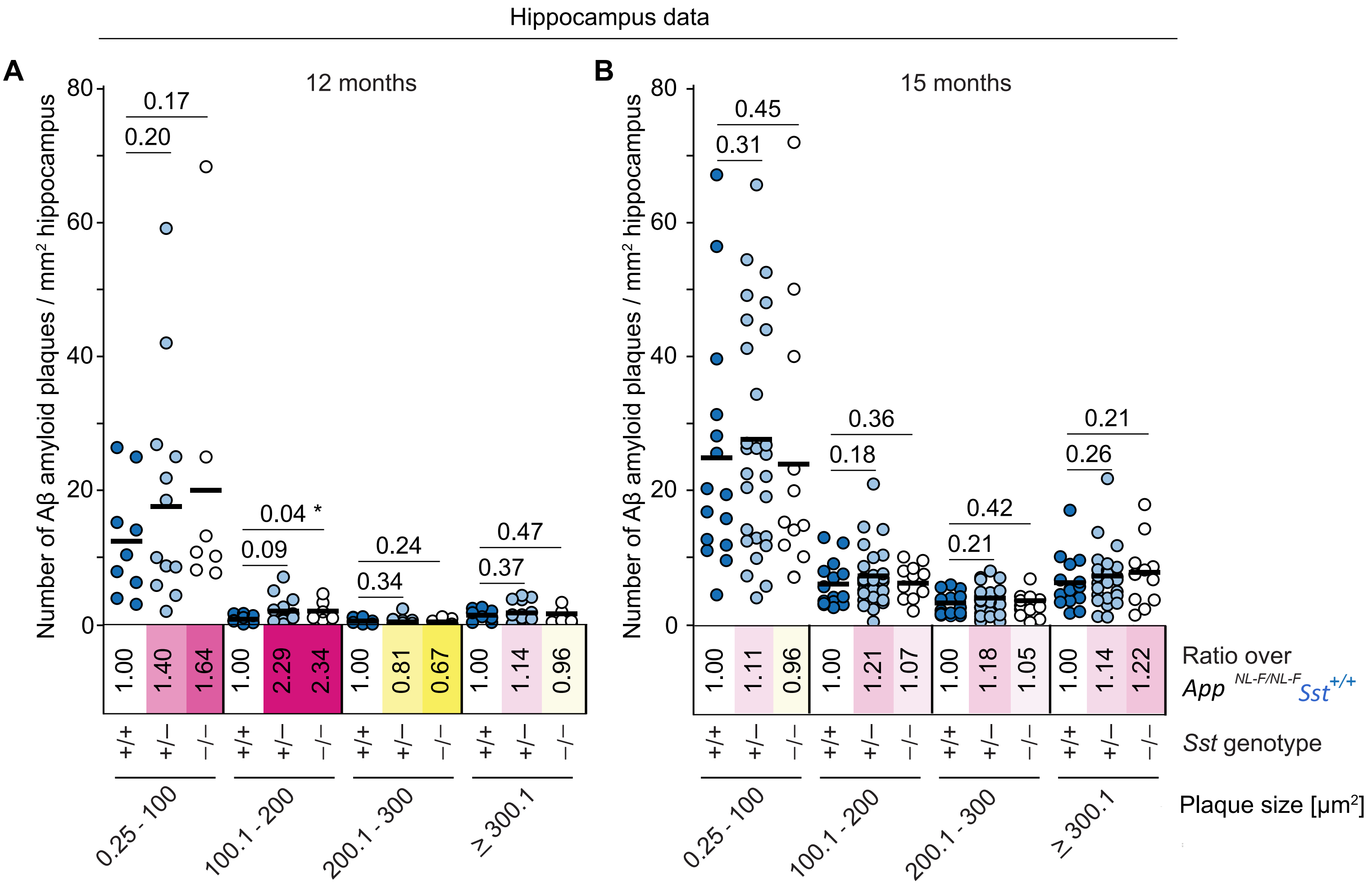

Supplementary Figure 3

Fig. 4A

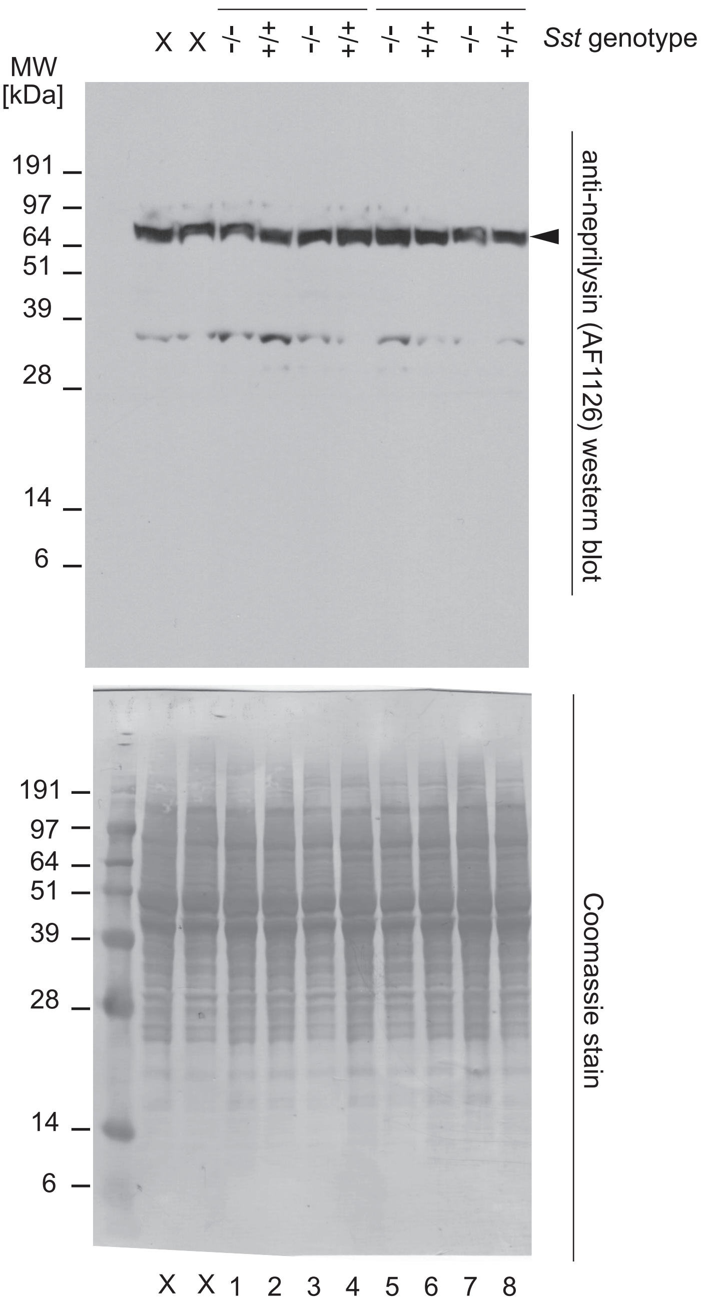

Fig. 5A

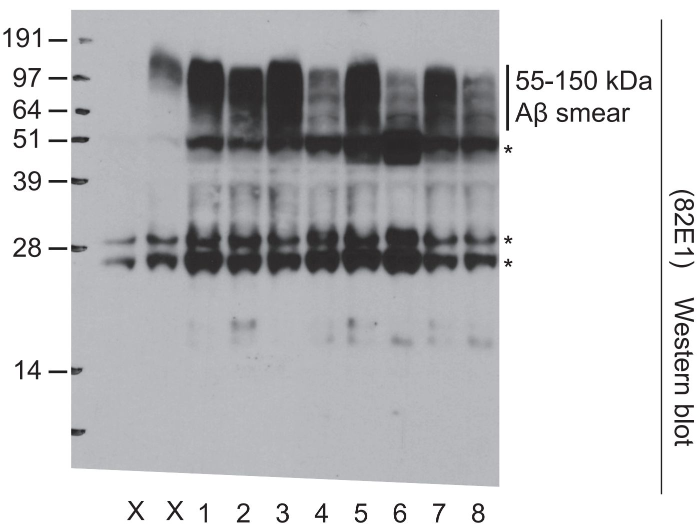

Fig. 5C

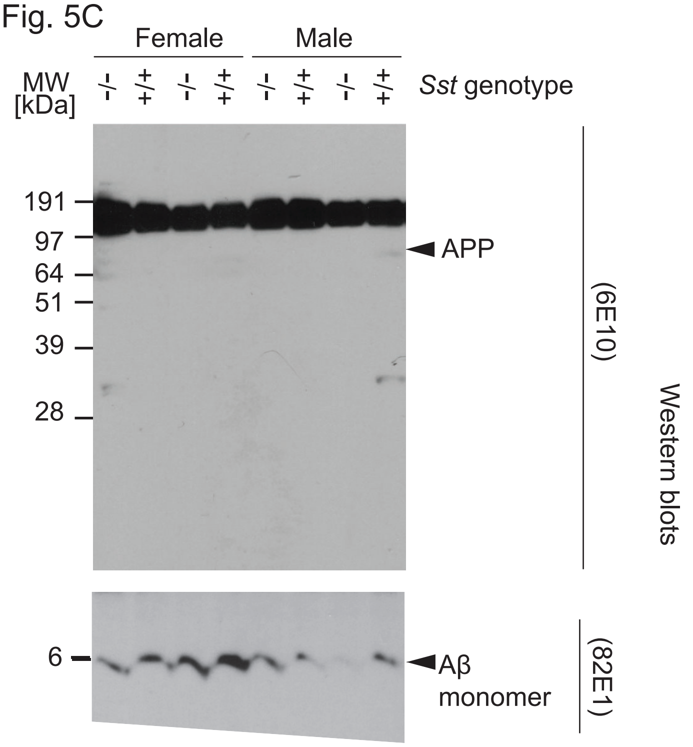

The western blot panels shown above and below were obtained after cutting the PVDF membrane horizontally and therefore do not extend to the full range of MW markers

Fig. 5D

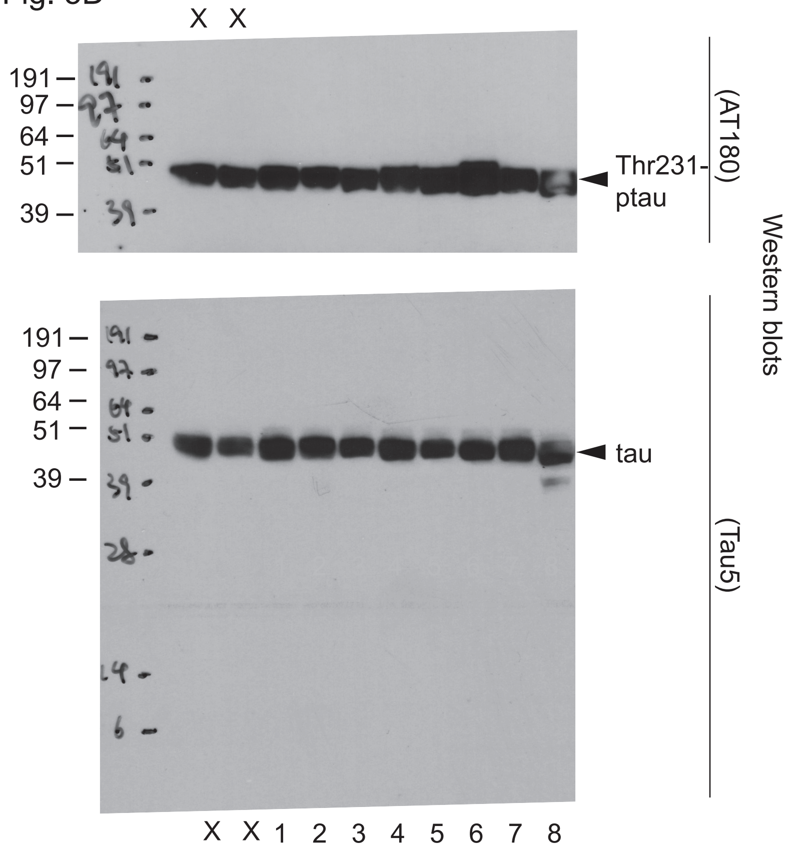

Supplement: Supplementary file 1 — Supplementary Figures. [file 41598_2023_29559_MOESM1_ESM.pdf]
